# Spatial and Temporal Control of Hyperthermia Using Real Time Ultrasonic Thermal Strain Imaging with Motion Compensation, Phantom Study

**DOI:** 10.1371/journal.pone.0134938

**Published:** 2015-08-05

**Authors:** Josquin Foiret, Katherine W. Ferrara

**Affiliations:** Department of Biomedical Engineering, University of California Davis, Davis, CA, United States of America; University of Minnesota, UNITED STATES

## Abstract

Mild hyperthermia has been successfully employed to induce reversible physiological changes that can directly treat cancer and enhance local drug delivery. In this approach, temperature monitoring is essential to avoid undesirable biological effects that result from thermal damage. For thermal therapies, Magnetic Resonance Imaging (MRI) has been employed to control real-time Focused Ultrasound (FUS) therapies. However, combined ultrasound imaging and therapy systems offer the benefits of simple, low-cost devices that can be broadly applied. To facilitate such technology, ultrasound thermometry has potential to reliably monitor temperature. Control of mild hyperthermia was previously achieved using a proportional-integral-derivative (PID) controller based on thermocouple measurements. Despite accurate temporal control of heating, this method is limited by the single position at which the temperature is measured. Ultrasound thermometry techniques based on exploiting the thermal dependence of acoustic parameters (such as longitudinal velocity) can be extended to create thermal maps and allow an accurate monitoring of temperature with good spatial resolution. However, *in vivo* applications of this technique have not been fully developed due to the high sensitivity to tissue motion. Here, we propose a motion compensation method based on the acquisition of multiple reference frames prior to treatment. The technique was tested in the presence of 2-D and 3-D physiological-scale motion and was found to provide effective real-time temperature monitoring. PID control of mild hyperthermia in presence of motion was then tested with ultrasound thermometry as feedback and temperature was maintained within 0.3°C of the requested value.

## Introduction

Non-invasive treatments based on mild temperature elevation (hyperthermia) have shown promising results in enhancing local drug delivery using temperature sensitive liposomes (TSL) [1–3] or for heat-activated gene delivery [4]. In these treatments, temperature is elevated and typically maintained between 39°C and 42°C to induce physiological changes for a short duration. Several studies have reported increased vascular permeability [5], increased blood flow [6], enhanced tissue oxygenation [7] and decreased pH [8] as a consequence of the increased temperature. Although mild hyperthermia has reversible effects on treated tissues, estimation of the thermal dose is important to minimize cell damage or death which increase as a function of both temperature and time [9]. The metric generally considered to define the threshold for thermal dose is the cumulative equivalent minutes at 43°C (CEM43). In this scenario, both spatial and temporal control of hyperthermia in the treated region is necessary to avoid irreversible thermal damage to surrounding healthy tissue.

High Intensity Focused Ultrasound (HIFU) is an efficient tool to deposit energy in deep tissues and have been used for treating uterine fibroids [10] and cancer [11, 12]. In this type of ablative therapy, tissue necrosis is induced by high temperature elevation (>55°C) in the targeted volume. Magnetic Resonance Imaging (MRI) is frequently used in the clinic for ablation planning and monitoring. MRI guidance has the advantage of offering excellent 3-D anatomical information as well as temperature maps on a broad range of temperature [13]. Understandably, real-time control of hyperthermia has been developed under MR guidance [13–16]. However, the main disadvantage of MRI is that its use is complex, costly [17] and available only in limited sites. Systems integrating ultrasound for both imaging and therapy have also been developed and are attractive due to their high spatial and temporal resolution, low-cost, and simplicity. Early systems have used mechanically-driven single-element transducers to create both the image and therapy. With the development of higher channel count arrays, new ultrasound imaging and therapy systems that are capable of real-time thermometry are advancing. Thus we examine the feasibility of employing ultrasound to control mild hyperthermia in the presence of motion.

With this perspective, several recent studies combining HIFU and ultrasound thermometry (UT) have demonstrated the potential for delivering mild hyperthermia under ultrasound control [18–21]. UT exploits the thermal dependence of acoustic parameters such as the backscattering coefficient [22] or the longitudinal speed of sound (SOS) [23–25] and has been shown to provide accurate temperature feedback along with good spatial resolution in the hyperthermia temperature range. The Change in Backscattered Energy (CBE) is based on the heat-induced change of the backscattered coefficient, which was shown to change nearly monotonically from 37°C to 50°C [22]. Similarly, the ultrasound echo-shift induced by the change in SOS was also shown to be proportional to the local temperature increase in the case of small temperature changes [23–25]. In this case, the parameter estimated is the axial gradient of the apparent displacement of the ultrasound speckle, the so-called thermal strain. As these techniques are based on the comparison of two imaging frames to detect the local change induced by heat (acquired before and during the treatment, or between successive frames during the treatment), they are sensitive to physiological motion such that small translations or deformations of the tissue are sufficient to create severe artifacts in the estimated temperature. For instance, a recent study on temperature monitoring in mice *in vivo* reported a standard deviation of 4.3°C without motion compensation, demonstrating the need for motion compensation in *in vivo* applications of ultrasound-based thermometry [21].

Physiological motion encountered in the clinic is primarily generated by cardiac and respiratory sources, the latter being the largest source of error in strain-based estimation [26]. In some regions (e.g. kidney and neck), cardiovascular motion is expected to dominate whereas in the liver and breast, respiration will be the main source of motion. Thus, for respiratory motion, periodicity on the order of seconds is reasonable. In the breast, motion can be minimized with appropriate patient positioning, however, motion is of a similar magnitude as in the liver [27]. Due to the relatively cyclic nature of physiological motion both in space and time, several methods for motion compensation have been successfully applied. Whenever possible, the transducer is oriented such that the physiological motion is within the imaging plane; with this orientation, motion correction is easily accomplished. However, in many practical situations, physiological motion is oriented both within and orthogonal to the imaging plane. Previous reports for motion in the liver with the probe oriented to minimize motion reported average peak-to-peak deviations in the axial dimension of 6.3 mm ± 3 mm, lateral deviations of 3.5 mm ± 1.5 mm and 3.8 mm ± 1.3 mm in the elevation dimension [28].

To maintain inter-frame correlation in the presence of motion, high-frame rate imaging was introduced [29] and coupled with adaptive spatial filtering [30]. Another method has demonstrated the ability to use multiple references frames acquired prior to treatment to compensate for motion [31]. A similar multi-reference frame approach using CBE was tested *in vivo* on tumor-bearing mice and was successful in reducing respiratory artifacts [21]. Displacement artifacts were similarly reduced using dynamic frame selection [32].

A novel approach focused on monitoring the changes in shear modulus has also shown good sensitivity to small temperature changes [33]. The technique, consisting of estimating the change in shear velocity (and thus shear modulus) with temperature, has also been shown to be fairly robust to tissue motion due to the very high frame rate needed to follow shear wave propagation. However, in pre-clinical studies [3], exciting and measuring shear wave propagation in small tumors and animals is problematic due to the relatively large wavelength. For our application of pre-clinical testing of new therapeutic strategies for treating cancer, a mm or sub-mm voxel is required in order to accurately assess the peak temperature within small tumors and therefore the shear wave approach is suboptimal. Further, the scale of physiological motion for the application addressed here (several mm in mice, increasing in larger animals) requires motion correction in order to accurately assess temperature changes on that same spatial scale.

In previous work by our group [34], mild hyperthermia was achieved using a proportional-integral-derivative (PID) controller with invasive thermocouple measurements. Despite providing excellent temperature control, the distribution of the temperature within the region cannot be mapped with this technique. The aim of this work is to provide real-time thermal maps in the presence of fully 3-D motion to actively control localized hyperthermia. Toward this end, we propose a method to monitor temperature based on real-time thermal strain imaging coupled with a multi-reference frame and motion correction algorithm [35]. Our specific goal in the work presented here is to explore the temperature estimation accuracy that can be achieved in the presence of increasing in-plane and out-of-plane motion. The method developed in this study was validated on a calibrated tissue-mimicking phantom. The sensitivity and robustness to motion of the technique was first tested with artificial physiological motion covering 2-D and 3-D displacements and both periodic and aperiodic motion patterns were investigated. We finally use the method to provide real-time temperature feedback and maintain hyperthermia in presence of motion through the use of a PID controller in scenarios that are relevant to pre-clinical therapeutic studies.

## Materials and Methods

### Experimental setup

**Fig 1:**
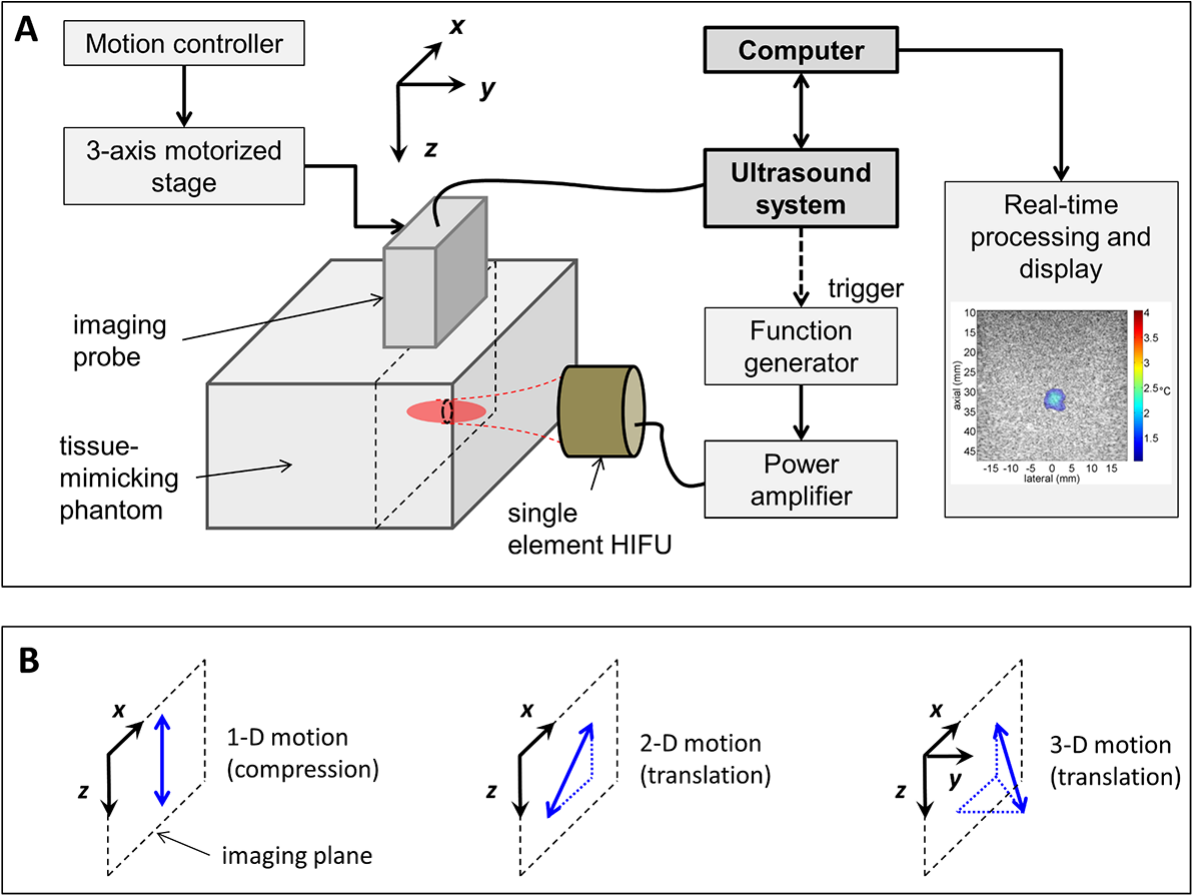
Experimental setup and tested artificial motions. (A) The diagnostic imaging array was placed above the tissue-mimicking phantom and physiological motion was simulated by moving the array using a 3-axis motorized stage. The single transducer element generating heat and the phantom were fixed. Ultrasound data were acquired on a programmable ultrasound system (Vantage 256, Verasonics) and processed and displayed in real-time using a dedicated computer. (B) Simulated cyclic motions: axial compression (1-D motion) and linear translations with in-plane (2-D motion) or out-of-plane displacement (3-D motion). Axial compression was generated by direct contact of the probe with the phantom.

A 5 MHz single-element transducer (IL0508HP, Valpey-Fischer, MA) was used to generate heat in an agarose-based tissue mimicking phantom. A 5 MHz 128-element imaging array (L7-4, Philips ATL, WA) was placed above the phantom with the imaging plane perpendicular to the heating transducer beam axis as depicted in Fig 1A. Physiological motion was simulated by moving the probe with a 3-D linear motorized stage remotely activated by a motion controller (ESP300, Newport Corporation, Irvine, CA). The choice of moving the imaging array rather than the phantom was to mimic an ultrasound treatment with real-time motion correction of the HIFU beam to track the target [36]. In this scenario, the ultrasound-generated heat was independent of the motion which facilitates evaluation of the quality of the temperature estimates. Radio-Frequency (RF) data were acquired with an ultrasonic acquisition system (Vantage 256, Verasonics, Redmond, WA) at a sampling frequency of 20 MHz.

The therapeutic transducer was driven by an arbitrary waveform generator (33500B, Agilent, Santa Clara, CA) and a 50-dB radiofrequency amplifier (325LA, ENI, Rochester, NY) and triggered by the Verasonics system. During the heating phase, the therapeutic pulse was activated immediately after the imaging acquisition. A minimum delay of 50 ms between the end of the heating pulse and the following imaging acquisition was included to prevent any ultrasound-induced motion of the phantom in the focal region while imaging. For the experiments reported in this work, the total acoustic power (TAP) of the HIFU transducer was 1.0 W (to generate a 4°C increase) or 1.6 W (7°C increase), as measured by a radiation force balance (UPM-DT-1AV, Ohmic Instruments Co., Easton, MD). For studies of open loop HIFU, ultrasound data were acquired over 80 s and HIFU was activated for 30 s (between *t*_ON_ = 10 s and *t*_OFF_ = 40 s). For studies of closed loop PID control, HIFU was applied for 260 s as detailed below.

Before heating, the acquisition frame rate was set to 20 Hz to collect the reference frames that will be used for motion compensation (see next Section). When thermometry was activated, the frame rate was lowered to 4 Hz for real time processing of thermal strain and display of the temperature map. Although the processing time for a single temperature map was approximately 30 ms, the frame rate was limited by the CPU which was also used to reconstruct the ultrasound images. The motion compensation algorithm was not impacted by this frame rate as discussed in the Discussion section.

Three artificial motion patterns were tested (Fig 1B): 1) linear axial compression within the imaging plane, 2) linear translation within the imaging plane (combining axial and lateral motion, referred to as 2-D diagonal) and 3) out-of-plane linear translation (combining axial, lateral and elevation motion, referred to as 3-D diagonal). For axial compression, mechanical strains of 5.0 and 7.5% were applied by the imaging probe on the phantom (axial displacement of 2.5 and 3.8 mm, respectively). For the 2-D diagonal motion, a total displacement of 4.2 to 7.1 mm in the imaging plane was tested.

Aperiodic (non-cyclic) motion was also investigated in order to evaluate whether the multi-reference based approach to motion correction required periodicity. The speed and displacement were changed randomly during the motion while remaining on the same diagonal trajectory. The total displacement was limited to 7.1 mm for this experiment (see S1 Fig).

Although most of the measurements presented in this work were acquired with a small temperature elevation (+4°C) to study the sensitivity of the technique to motion, a higher temperature elevation (+7°C) was also investigated for the 2-D motion. This case corresponds to the upper limit for hyperthermia on mice (from a body temperature of 36°C to 43°C).

Finally, the 3-D diagonal motion extended the 2-D motion by adding motion in elevation for a total displacement ranging from 5.2 to 8.7 mm. The parameters of the displacements induced for each experiment are summarized in Table 1. For all of the 1-D and 2-D experiments with cyclic motion, the period was set to 2 s. For 3-D experiments, the period was decreased to 4 s in order to measure temperature changes related to out-of-plane motion.

**Table 1:** Parameters used for the different experiments mimicking an artificial physiological motion.

	Displacement (mm) along axis			Total displacement (mm)	Maximum velocity (mm/s)	Number of reference frames
	*x*	*y*	*z*			
**1-D compression**	2.5	0	0	2.5	2.5	30
	3.8	0	0	3.8	3.8	40
**2-D diagonal**	3	0	3	4.2	4.2	50
	4	0	4	5.7	5.7	60
	5	0	5	7.1	7.1	70
**3-D diagonal**	3	3	3	5.2	2.6	50
	4	4	4	6.9	3.5	60
	5	5	5	8.7	4.4	70

The periodicity of the motion was fixed to 2 s for 1-D and 2-D experiments such that the maximum velocity reached during the motion was equal to the total displacement value in mm/s. For the 3-D experiments, the periodicity of the motion was fixed to 4 s. The number of reference frames indicates the number of frames acquired before thermal strain estimation. The axes *x*, *y* and *z* are associated with the axes shown in Fig 1.

All experiments were performed in pure degassed water at room temperature. Temperature estimation was also applied on data acquired without motion for comparison and validation purposes.

### Tissue mimicking phantom

An agarose-based evaporated-milk phantom with properties similar to soft tissue and described elsewhere [37] was used in this study. The phantom consisted of fat-free evaporated milk (Carnation, Nestle USA, Inc., Solon, OH), Dulbecco’s phosphate-buffered saline (DPBS, Mediatech, Inc., Manassas, VA), and *n*-propanol (Sigma-Aldrich Company LLC) with a volume ratio of 46:50:4. Agarose powder (OmniPur Agarose, EMD Chemicals, Inc., Gibbstown, NJ) and silicon carbide for acoustic scattering (HSC1200, d_50_ = 6 μm, Superior Graphite, Chicago, IL) were added at a weight/total-volume ratio of 2% and 1.5%, respectively. The phantom density was 1.05 g/cm^3^ and the speed of sound was 1540 m/s at 22°C. The temperature dependence of the speed of sound was 1.3±0.5 m/s/°C [37].

### Temperature estimation with motion compensation

#### Background

Thermal strain imaging is based on the time shift estimation of backscattered echoes induced by a local change in tissue temperature [**24**, **25**, **38**]. Temperature dependence of the speed of sound (SOS) and thermal expansion of the tissue are the two phenomena at the origin of the echo time shift. These combined effects lead to local speckle modification in ultrasound images when a temperature change occurs. Over a limited temperature range, the apparent displacement in the lateral position *x* and depth *z* in the imaging plane is related to the temperature elevation by the relationship below [**25**]:

$$\Delta T\left(x,z\right)=\frac{c\left(T_{0}\right)}{2}\frac{1}{\alpha -\beta }\frac{\partial }{\partial z}\left(\tau \left(x,z\right)\right)$$ (1)

where Δ*T*(*x*,*z*) is the temperature elevation, *τ*(*x*,*z*) the apparent time shift of the echoes, *c*(*T*_0_) the initial speed of sound at baseline temperature *T*_0_, *α* the linear coefficient of thermal expansion and *β* = 1/*c*·∂*c*/∂*T* the coefficient related to the change of speed of sound with temperature. For small temperature increases and for water-bearing tissues, the effect of thermal expansion is small compared to speed of sound changes and can be reasonably neglected [24, 38]. The term *k* = 1/(*α*-*β*) is material dependent and is assumed to be constant with temperature. Therefore, as the change in SOS is seen as an apparent or virtual displacement of the scatterers along the ultrasound beam axis, local temperature changes can be estimated by tracking the apparent displacement of the speckle. The relationship between temperature change and thermal strain is then expressed as:

$$\Delta T\left(x,z\right)=k\frac{\partial }{\partial z}\left(\Delta d\left(x,z\right)\right)$$ (2)

with Δ*d*(*x*,*z*) = *c*(T_0_)/2·*τ*(*x*,*z*). The derivative of Δ*d* with respect to *z* is the so-called thermal strain expressed in %. For water bearing tissues, *k* has been reported to be -1200°C for beef muscle [25] or -1405°C for turkey muscle [33].

#### Thermal strain estimation

**Fig 2:**
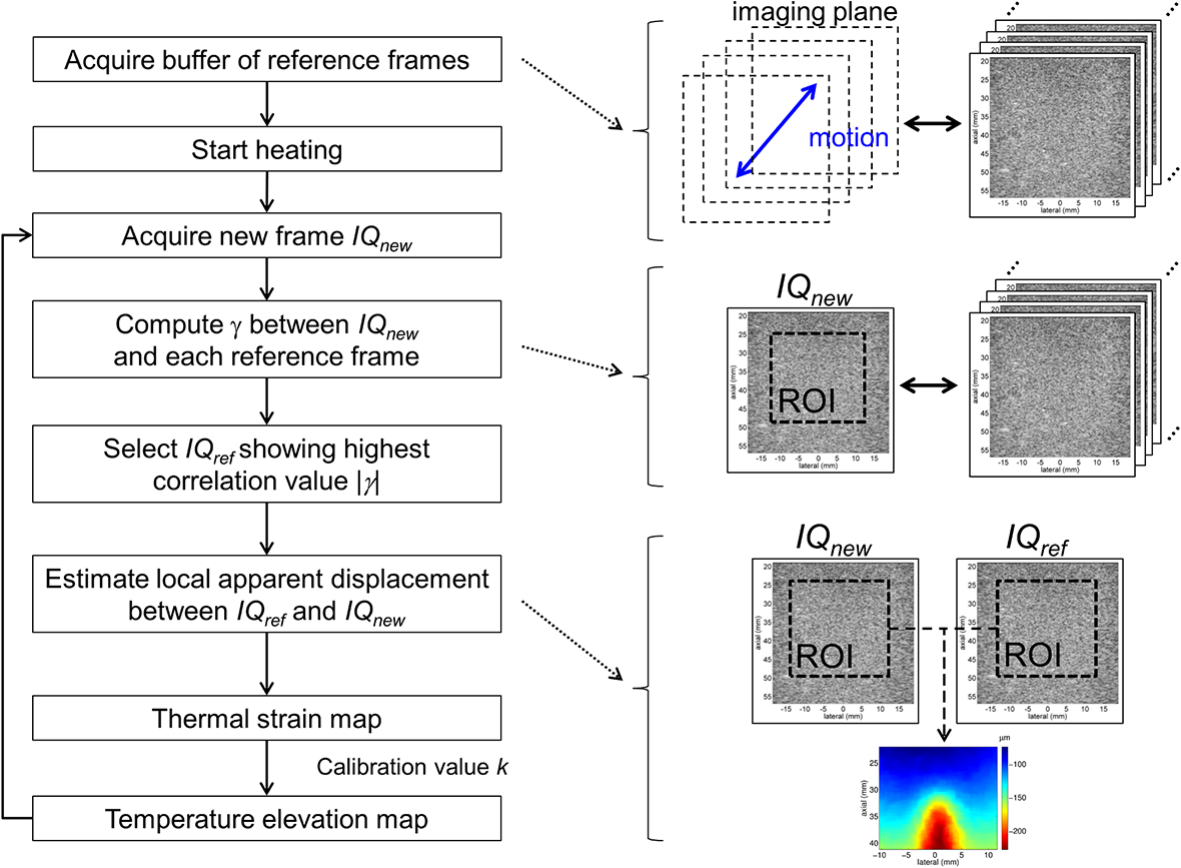
Block diagram of the motion compensation algorithm. Temperature elevation is calculated from thermal strain using a buffer of reference frames to compensate for tissue motion.

Apparent displacement was calculated in real time using two-dimensional speckle tracking routines. The algorithm tracks speckle changes between continuously acquired frames (during heating) and a reference frame chosen from a set of frames acquired at baseline temperature (before heating) in a defined region of interest (ROI). The word ‘frame’ is referring here to the In-phase/Quadrature (IQ) beamformed complex data. To compensate for tissue motion, the reference frame was chosen within a buffer of N_ref_ reference frames that samples the motion in space as proposed in [**31**]. The measurement was thus divided into two successive sequences: first, acquisition of the N_ref_ reference frames before heating, then thermal strain estimation, as shown in **Fig 2**.

During acquisition of the references frames, spatial sampling of motion was improved by evaluating the correlation of each new frame with the reference frames previously recorded. Complex two-dimensional cross-correlation was computed using the expression:

$$\gamma =\frac{\sum_{m=-M/2}^{M/2}\sum_{n=-N/2}^{N/2}\left(IQ_{ref}^{*}\left(m_{0}+m,n_{0}+n\right)\cdot IQ_{new}\left(m_{0}+m,n_{0}+n\right)\right)}{\sqrt{\sum_{m=-M/2}^{M/2}\sum_{n=-N/2}^{N/2}{\left|IQ_{ref}\left(m_{0}+m,n_{0}+n\right)\right|}^{2}}\sqrt{\sum_{m=-M/2}^{M/2}\sum_{n=-N/2}^{N/2}{\left|IQ_{new}\left(m_{0}+m,n_{0}+n\right)\right|}^{2}}}$$ (3)

where IQ_ref_ is the reference frame in the buffer, IQ_new_ is the most recently acquired frame, M and N are the number of samples in the ROI in the axial and lateral dimensions, and (*m*_0_, *n*_0_) are the indices of the center of the ROI. The symbol * denotes complex conjugation. Each acquired frame was considered as a new reference frame only if the magnitude of the correlation values, |*γ*|, as calculated with all of the reference frames, was below a threshold value of 0.95. A minimum of 30 frames was recorded prior to hyperthermia. Tissue heating and monitoring of temperature start once spatial sampling of the motion was considered sufficient (i.e. each new frame was correlated to at least one of the reference frames).

During the thermometry sequence, for each newly acquired frame, the algorithm identified the best matching reference frame within the buffer. The magnitude of the correlation value from Eq (3) was computed with all of the N_ref_ references frames and the frame showing the highest value was selected for speckle tracking.

The local apparent displacement was then computed by tracking the displacement of small two-dimensional kernels over larger search regions in the reference frame. The complex cross-correlation between the reference and current frame was computed using the expression [39]:

$$\gamma_{m_{0},n_{0}}\left(i,j\right)=\frac{\sum_{m=-K/2}^{K/2}\sum_{n=-L/2}^{L/2}\left(IQ_{new}\left(m_{0}+m,n_{0}+n\right)\cdot IQ_{ref}^{*}\left(m_{0}+m+i,n_{0}+n+j\right)\right)}{\sqrt{\sum_{m=-L/2}^{K/2}\sum_{n=-K/2}^{L/2}{\left|IQ_{new}\left(m_{0}+m,n_{0}+n\right)\right|}^{2}}\sqrt{\sum_{m=-K/2}^{K/2}\sum_{n=-L/2}^{L/2}{\left|IQ_{ref}\left(m_{0}+m+i,n_{0}+n+j\right)\right|}^{2}}}$$ (4)

where (*m*, *n*) refers to the pixel position in the kernel area, (*i*, *j*) is the position of the kernel in the searching range, K and L are the number of samples in the kernel in the axial and lateral dimensions. The indices (m_0_, n_0_) indicate the center of the kernel. Assuming A and B are the number of samples in the search area in the axial and lateral dimensions (with A≥K and B≥L), *γ*(*i*, *j*) will have (A-K+1)×(B-L+1) values. The difference between the position of the maximum of |*γ*(*i*, *j*)| and its central element ((A-K+1)/2,(B-L+1)/2), assuming A, B, K and L are odd integers) gives the axial and lateral lag of the kernel, respectively at the indices *i*_lag_ and *j*_lag_. A sub-sample estimate of the axial lag was then achieved by searching for phase cancellation in the cross-correlation function at the lateral lag *j*_lag_ around its maximum [40]. The kernel size and search region were chosen to be 5λ×5λ (1.5×1.5 mm^2^) and 9λ×9λ (2.8×2.8 mm^2^) respectively, where λ is the wavelength of the imaging pulse. The choice of the kernel size was made in order to provide sufficient spatial resolution without introducing sensitivity to noise. The kernels are selected at a 1λ interval (0.3 mm) in both the axial and lateral dimensions giving an overlap of 80%. Thus, the output apparent displacement map has an isotropic pixel size of 1λ×1λ (0.3×0.3 mm^2^).

**Fig 3:**
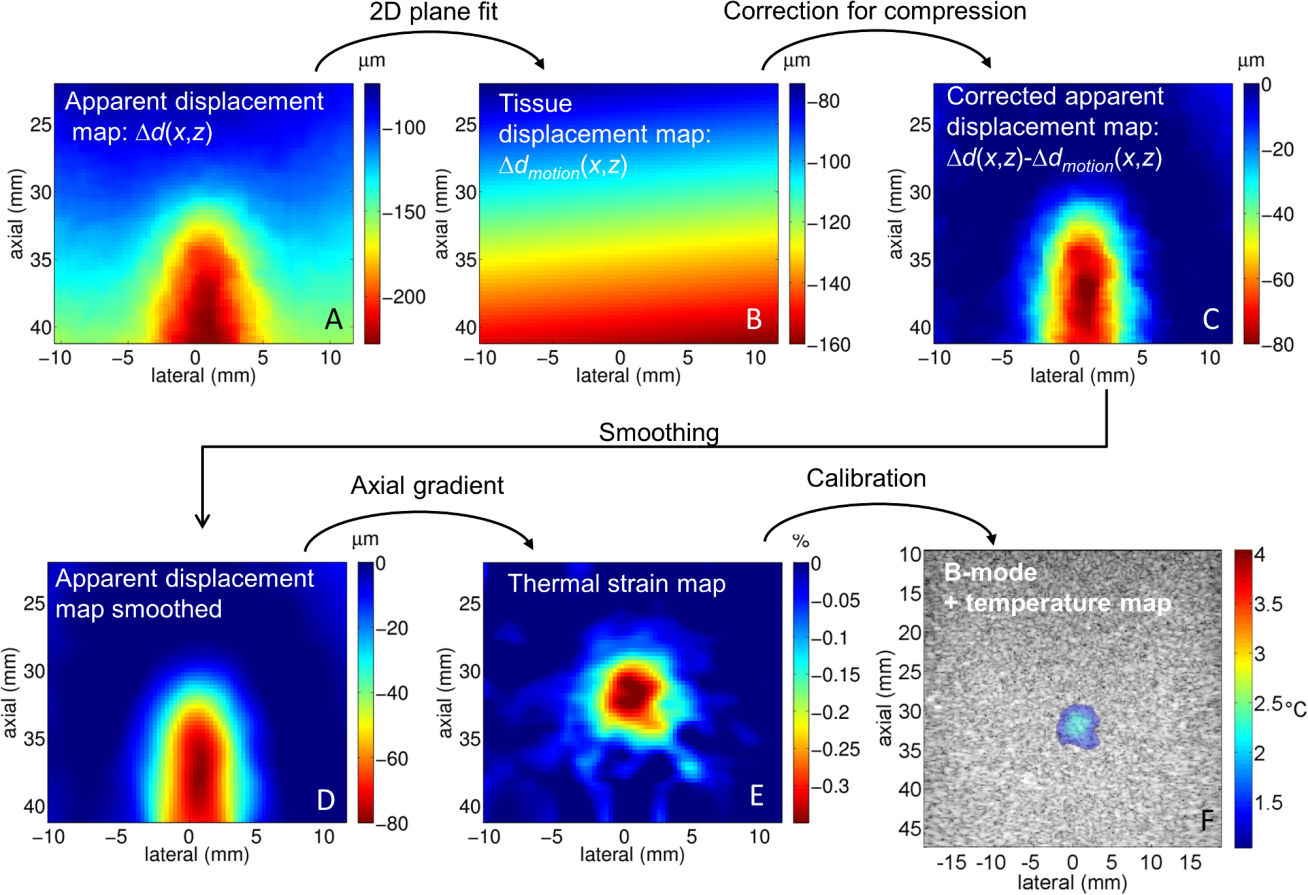
Processing steps between the apparent displacement map and the final temperature map. First the raw apparent displacement map was calculated (A), and then the equation of a plane was fit outside of the heated area to estimate the compression of the tissue (B). The resulting map was then subtracted from the raw apparent displacement map (C). The corrected map was then smoothed (D) and the axial gradient was calculated to get the thermal strain map (E). Using the calibration value, the temperature map is superimposed on the B-mode image (F). This example presents the correction applied for a periodical linear axial compression inducing a 7.5% mechanical strain on the phantom.

An estimate of the axial lag induced by tissue motion was calculated and subtracted from the shift map leaving only the apparent displacement induced by the change in temperature. During this step, a plane was fitted to the axial displacement map by estimating the coefficients *a*, *b* and *c* of the equation:

$$\Delta d_{motion}\left(x,z\right)=ax+bz+c$$ (5)

and then subtracted from the displacement map as depicted in Fig 3. During this process, the heated area was not included in the calculation. This process particularly helps to differentiate tissue motion from apparent displacement in the case of axial compression. The map was then successively smoothed with a 1-D Savitzky-Golay filter [41] in the lateral and axial dimensions with window sizes of respectively 11λ and 17λ (3.4 and 5.2 mm). The thermal strain map was finally obtained by differentiating the apparent displacement map in the axial dimension.

To illustrate the different steps involved in the thermal strain estimation, one can refer to the example depicted in **Fig 3**. The non-corrected displacement map (**Fig 3A**) illustrates both the effect of the mechanical motion and the apparent shift due to the local change in temperature. When the displacement due to tissue motion was identified (**Fig 3B**), the change caused by temperature elevation can be isolated (**Fig 3C and 3D**) and thermal strain and temperature correctly estimated (**Fig 3E and 3F**).

All processing was done using Matlab (The Mathworks Inc., Natick, MA) and tracking routines were implemented in C language as MEX (Matlab EXecutable) functions.

### Calibration

Prior to measurements with motion, the parameter *k* = 1/(*α*-*β*) was measured by comparing estimated strain with invasive temperature measurements using a 30-gauge × 13-mm T-type needle thermocouple (HYP-1, Omega Engineering, Inc., Stamford, CT). As the thermocouple was encased in a steel needle, it produced negligible temperature artifacts. For the HYP-1 thermocouple, a previous study [19] reported measured artifacts of 0.08 ± 0.02°C *in vitro* and 0.04 ± 0.02°C *in vivo*. Thus, the thermocouple reading was considered as the true temperature.

### Temperature control

After testing and validating the algorithm with different types of motion, the strain-based temperature was utilized for real-time control of the thermal dose, following previous work done by our group [34]. To this end, the estimated temperature was fed to a proportional-integral-derivative (PID) controller to regulate and maintain the temperature at a requested value. The input of the PID controller was the error between the set temperature and the temperature estimated from thermal strain. The output was converted into a duty factor value and sent to the function generator driving the HIFU transducer. The algorithm is summarized as [34, 42]:

$$DF_{i}=K_{P}e_{i}+\sum_{i}K_{I}\Delta t\left(\frac{e_{i}+e_{i-1}}{2}\right)+\frac{K_{D}}{6\Delta t}\left(e_{i}-e_{i-3}+3\left(e_{i-1}-e_{i-2}\right)\right)$$ (6)

with *DF* the duty factor, *e* the temperature error, *Δt* the sampling interval, *K*_*P*_, *K*_*I*_ and *K*_*D*_ the proportional, integral and derivative gains, respectively. The subscript *i* indicates the time index. After thorough testing, the best values for *K*_*P*_, *K*_*I*_ and *K*_*D*_ were found to be 2.0, 0.0002 and 1.0, respectively, with a sampling interval of 0.25 s. The maximum duty factor was limited to be 40% which corresponds to 1 W TAP.

A 3λ×3λ (0.9×0.9 mm^2^) region of interest centered on the focal spot was used to assess temperature. After acquiring the reference frames, the PID controller was activated and the requested temperature value was +4°C above the ambient value and maintained for 260 s. The set point temperature was chosen to follow a rising exponential with a time constant of 7 s in order to prevent ringing in the controller. With this time constant, the time to reach +4°C was approximately 30 s giving an average heating rate of 0.13°C/s and the steady-state value was retained for 230 s. The 2-D translational motion (in plane) with the displacement values reported in Table 1 was then tested. The frame rate was set to 20 Hz to acquire the reference frames and subsequently decreased to 4 Hz for thermal strain estimation.

## Results

### Reference temperature

**Fig 4:**
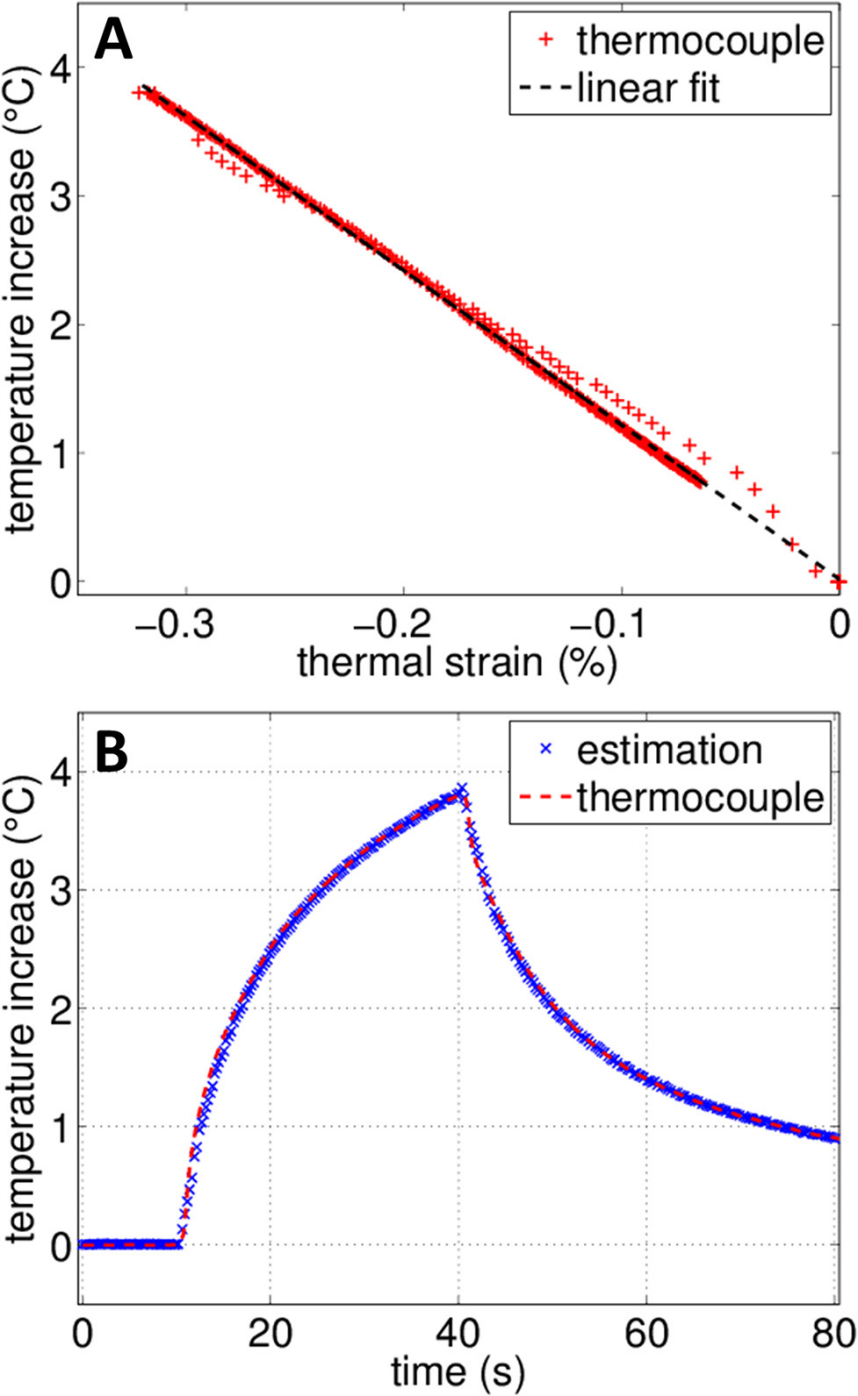
Calibration measurement for the tissue-mimicking phantom. (A) Calibration of the material dependent *k* value for the tissue-mimicking phantom by comparing temperature elevation measured by the thermocouple and corresponding thermal strain. (B) Comparison between temperature changes estimated from thermal strain after calibration and thermocouple measurement.

To evaluate the accuracy of the strain-based temperature without motion, thermal strain was estimated on the phantom and compared to thermocouple measurements. Thermal strain was averaged over a ROI of 3λ×3λ (0.9×0.9 mm^2^) centered on the heated region. The parameter *k* = 1/(*α*-*β*) was estimated to be -1220 ± 20°C for the phantom material with high correlation (R^2^ = 0.99) between estimated thermal strain and thermocouple measurements (Fig 4A). This value is similar to reported values for *ex vivo* muscle [25, 33]. The maximum temperature elevation after 30 s of heating was 3.8°C as measured by the thermocouple. After calibration, the strain-based temperature estimation matched the thermocouple measurement with an absolute error less than 0.1°C, as shown in Fig 4B. The temperature estimation without motion was thus considered as the reference temperature in the following sections due to its high correlation with thermocouple readings.

### Temperature estimation in presence of motion

The same HIFU parameters (heating time of 30 s, 1 W TAP) were used while generating artificial motion by moving the imaging array as described in Section II.A. The number of reference frames used for each motion is reported in Table 1 and was adapted to the motion amplitude (i.e. larger displacement requires more references frames for proper spatial sampling). At the frame rate of 20 Hz, acquisition of the references frames was completed within 10 to 30 seconds for the motion reported in this study.

**Fig 5:**
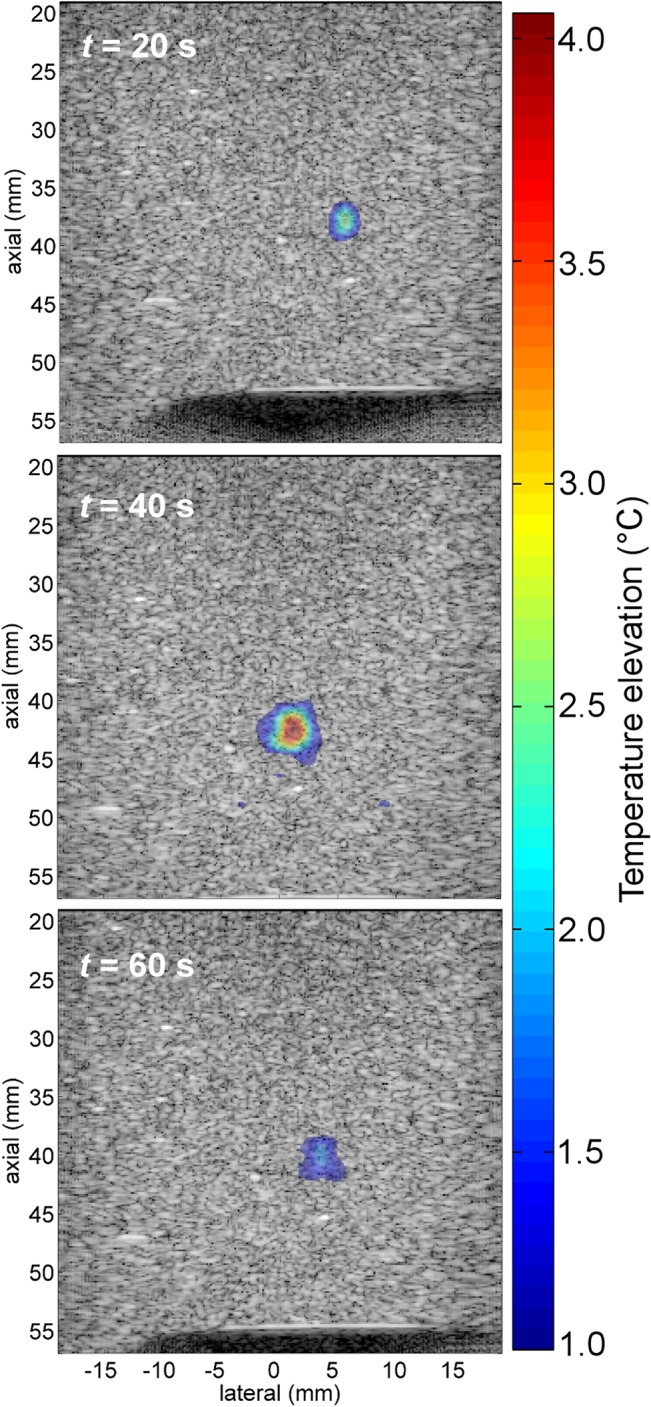
Temperature map and ultrasound imaging. Temperature map is superimposed on B-mode image at different times for 2-D diagonal motion with a total displacement of 7.1 mm. HIFU was activated for 30 s (between *t*_ON_ = 10 s and *t*_OFF_ = 40 s. To enhance visibility, pixels with a temperature elevation below 1˚C are not displayed on the image.

**Fig 6:**
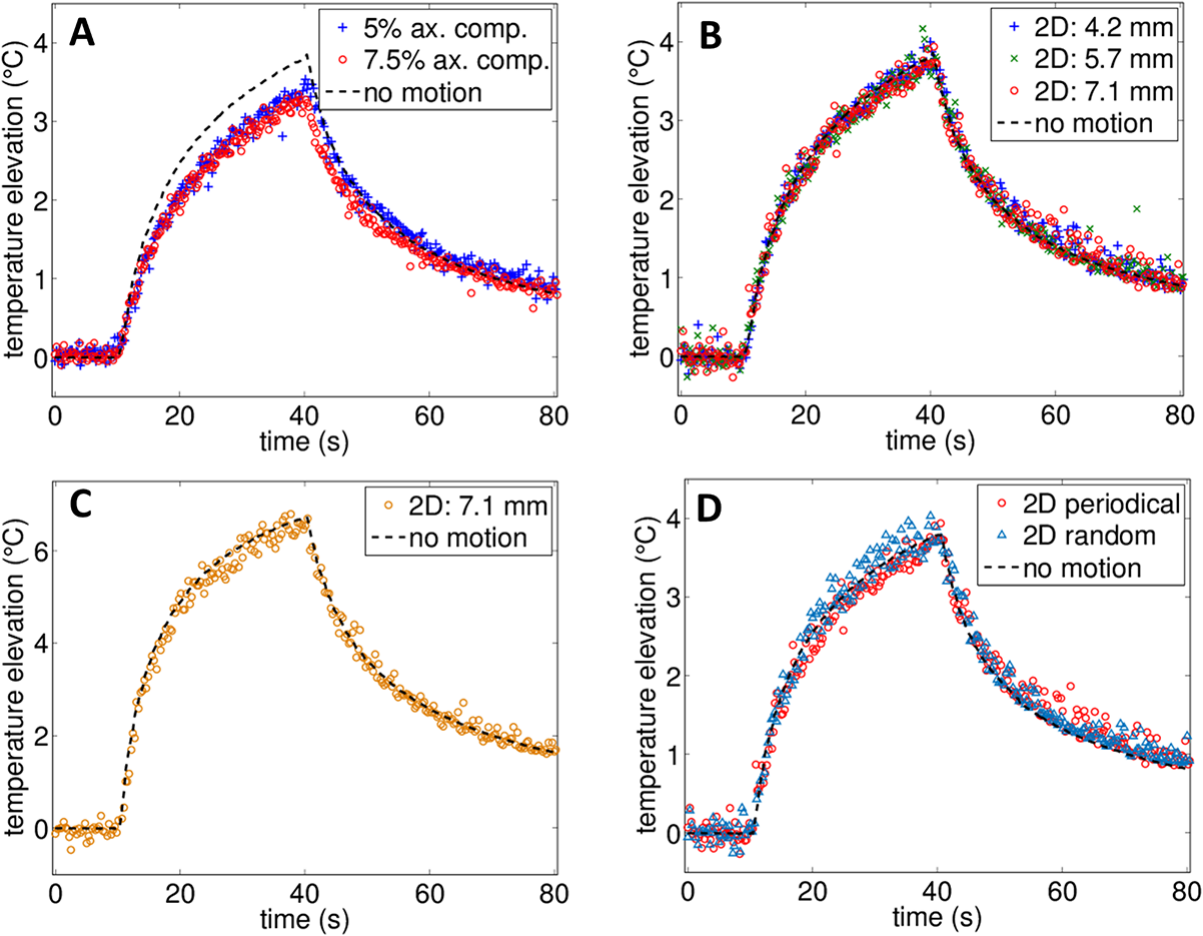
Comparison of estimated temperature elevation with and without artificial 1-D and 2-D physiological motion. (A) Axial compression in 1-D (ax. comp.), (B) in-plane diagonal motion (2-D) in the presence of a 4°C increase, and (C) in-plane diagonal motion (2-D) in presence of a 7°C increase. (D) Comparison of temperature estimation in presence of periodic 2-D motion (2-s period) and aperiodic motion. For the aperiodic motion, the displacement was diagonal and bounded to a maximum displacement of 7.1 mm but the speed and position was varied randomly during the motion. For all of the measurements, HIFU was applied between *t* = 10 s and *t* = 40 s. Movies of the strain-based temperature map superimposed on the B-mode image in the presence of axial compression and 2-D diagonal motion are provided in the supplementary materials S1 and S2 Movies, respectively.

An illustration of real-time temperature monitoring combining ultrasound imaging with a temperature map is given in Fig 5. The real-time display provides both spatial and temporal information regarding the temperature distribution. Tracking the center of the heated region gives the estimated temperature elevation as a function of time which is displayed in Fig 6. Temperature was averaged over a ROI of 3λ×3λ (0.9×0.9 mm^2^). Movies of the associated sequences are provided in the supplementary materials S1, S2 and S3 Movies. For all measurements in the presence of motion, a high correlation was obtained with the reference measurement with a clear separation between the heating and cooling phases. Differences between the experiments result from the deformation of the phantom (axial compression) or the motion of the imaging array (3-D motion).

When periodic axial compression was applied (Fig 6A), as the acoustic energy was spread over a wider area due to the motion, and the peak temperature elevation was smaller than the static case. The estimated maximum temperature elevation was 3.5 and 3.3°C for the 5 and 7.5% axial compressions, respectively, compared to the 3.8°C elevation of the reference measurement. The root-mean-square error (RMSE) generated by the motion can be estimated with sequences without heating by comparing the estimated temperature to an expected 0°C value. The RMSE was then found to be 0.12°C and 0.14°C (5% and 7.5% compression respectively). By omitting the correction for axial variation applied on the apparent displacement (Fig 3B and 3C), the error was 2 times larger, reaching 0.24 and 0.28°C respectively.

For the 2-D diagonal motion (Fig 6B, 6C and 6D), we can directly compare the result with the reference measurement. The estimated temperatures were similar to the reference case and the largest RMSE occurred for motion with the largest displacement (7.1 mm). The RMSE was respectively 0.10°C, 0.12°C and 0.14°C for the 4.2 mm, 5.7 mm and 7.1 mm displacement experiments, respectively. Similarly, the spatial extent of the heated area was comparable for all measurements. At the highest temperature elevation (*t* = 40 s), the area over which temperature was elevated by more than 1°C (as displayed in Fig 5) was 23.9, 25.2 and 23.4 mm^2^ which was comparable to the 27 mm^2^ measured for the reference case.

The temperature elevation was also accurately estimated with a higher imposed elevation of 7°C (Fig 6C) which corresponds to the upper limit for mild hyperthermia on mice (from 36°C to 43˚C). In this case, the RMSE was 0.18°C which is similar to the value obtained for the lower 4°C elevation.

Finally, as the motion compensation algorithm relies on the use of multiple reference frames, the technique is not sensitive to the periodicity or speed of the motion but rather on the correlation between frames as demonstrated in Fig 6D. When the motion followed the trajectory of the 2-D diagonal displacement but the speed and extent of the motion were randomly varied (with the displacement limited to a maximum of 7.1 mm), no significant differences were found in the temperature estimation. The RMSE was 0.14°C which was equal to the value found for the periodical experiment.

**Fig 7:**
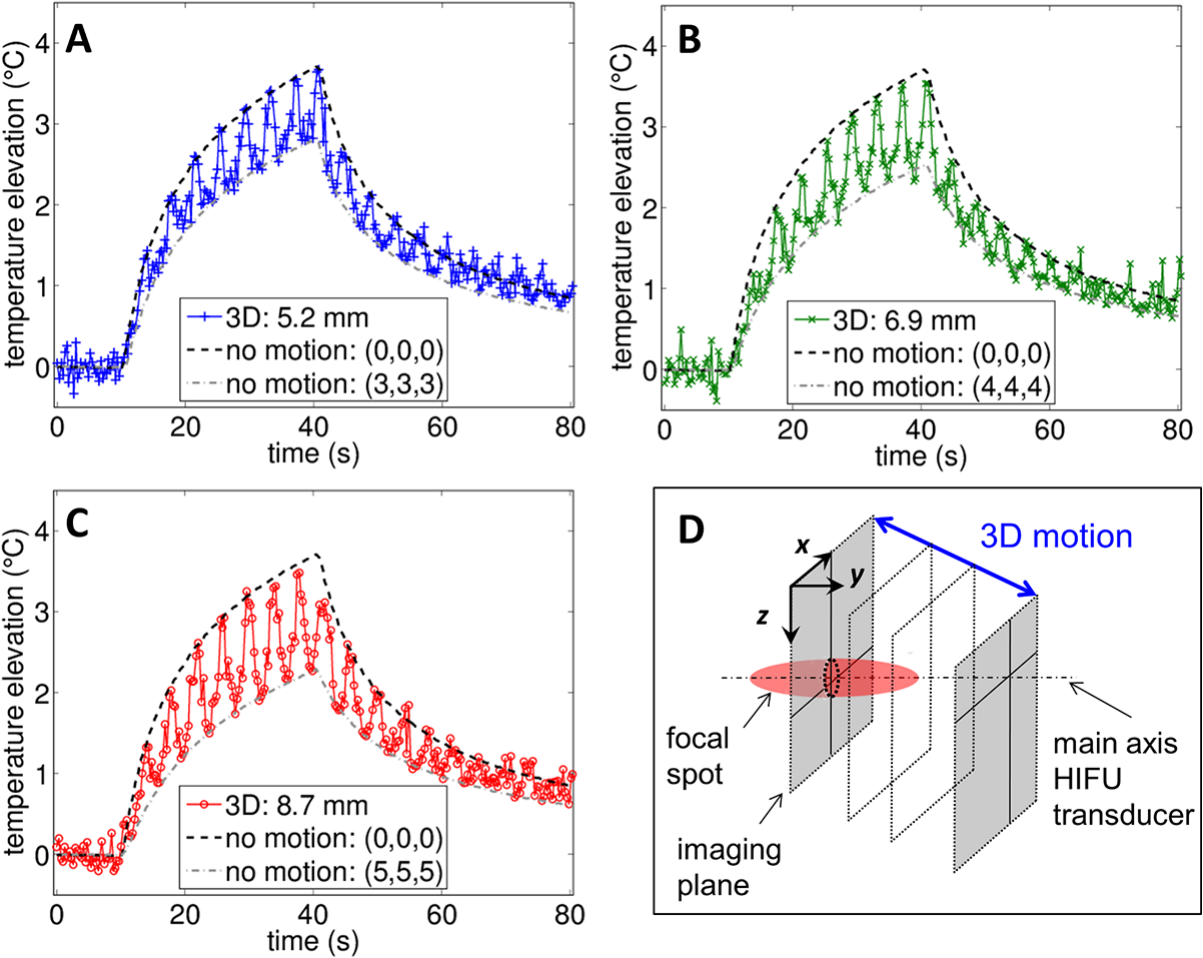
Comparison of estimated temperature elevation with and without 3-D artificial physiological motion. Temperature elevation measured for the 3-D diagonal motion with a total displacement of (A) 5.2 mm, (B) 6.9 mm, and (C) 8.7 mm. For all three measurements, HIFU was applied between *t* = 10 s and *t* = 40 s. (D) Lower temperature values are associated with out-of-plane periodicity as the probe moves away from the focal spot. A movie of the strain-based temperature map superimposed on the B-mode image in the presence of 3-D diagonal motion is provided in the supplementary materials (S3 Movie).

Lastly, the results for 3-D motion are presented in Fig 7A, 7B and 7C. Compared to the previous 1-D and 2-D motion, cyclic oscillations are clearly observed in the temperature estimation resulting from the back and forth motion of the probe as summarized in Fig 7D. As the probe undergoes motion, the imaging plane was moved away from the heated region (fixed in space) and the temperature elevation decreased accordingly. The upper bound of the curve followed the temperature estimated when the imaging array was right above the focal spot while the lower bound followed the temperature estimated when the imaging array was at the most distant point in elevation (along *y* axis). Thus higher out-of-plane displacement led to greater temperature oscillations. This result shows that the temperature estimation is able to track the out-of-plane motion. Without heating, the RMSE generated by the 3-D motion was estimated to be 0.08°C, 0.10°C and 0.11°C for a total displacement of 5.2, 6.9 and 8.7 mm, respectively.

As demonstrated by the temperature estimation results, the use of several reference frames efficiently tracks motion and maintains correlation between frames. Here, the metric used to assess the use of the multi-reference frame approach compared to a single frame is the percentage of frames that can be processed assuming the correlation magnitude in the ROI exceeds a threshold value, chosen to be 0.8 (which is the value ensuring correct temperature estimation). Using this metric, 100% of the frames can be processed for all 1-D, 2-D and 3-D motions with the multi-reference frames method. Using a single reference frame, this number drops to 10.0 and 6.0% for the 1-D motion (respectively 2.5 mm and 3.8 mm displacements), 4.2, 3.9 and 2.1% for the 2-D motion (respectively 4.2 mm, 5.7 mm and 7.1 mm displacements), 6.3, 3.8 and 3.2% for the 3-D motion (respectively 5.2 mm, 6.9 mm and 8.7 mm displacements). Therefore, without the multiple reference frames, only a small fraction of the sequences can be accurately processed.

**Fig 8:**
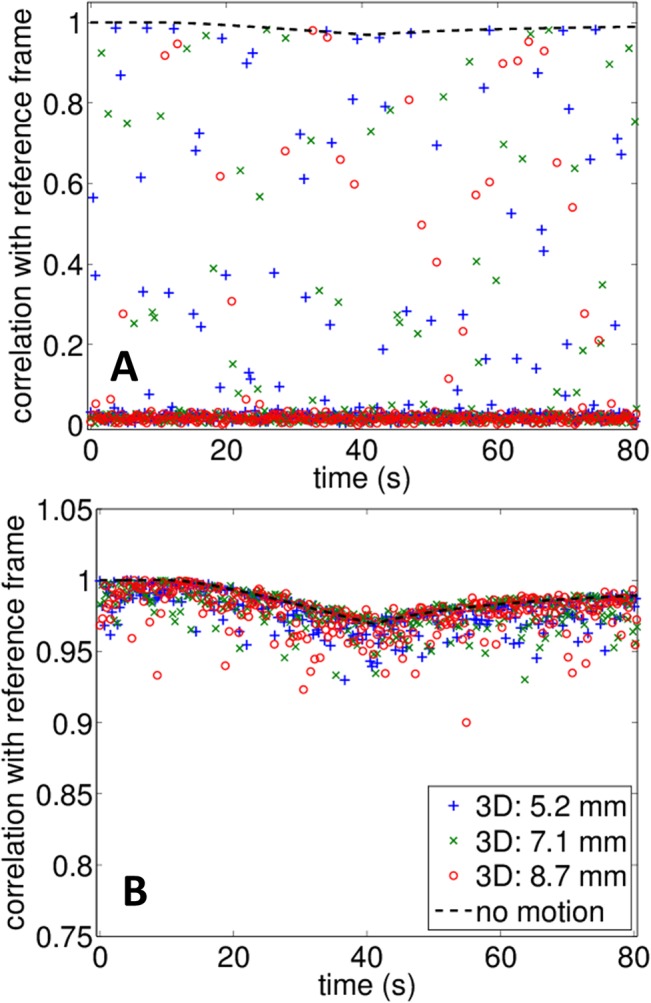
Correlation magnitude between frames acquired during the thermometry sequence and the matching reference frames for 3-D motion measurements as calculated over the entire ROI. (A) With a single reference frame and (B) with the proposed multi-reference frame method. With a single reference frame, only a fraction of the sequence shows high correlation and can be processed for temperature estimation. With the proposed method, correlation is maintained during the whole sequence.

To reflect this result, the magnitude of the correlation over the ROI is shown at each time point for the 3-D motion measurements in Fig 8. If a single reference frame is used, the correlation magnitude between the current and reference frame approaches zero and thermal strain cannot be calculated (Fig 8A). With the multi-reference frame method (Fig 8B), the correlation magnitude exceeded 0.85 and the mean temperature elevation estimate acquired over time was similar to the experimental results without motion, even for experiments in which the probe was translated by 8.7 mm. This result is consistent with decorrelation measurements performed with the L7-4 imaging array (see S2 Fig) where complete decorrelation (correlation value <0.1) between two frames was measured for a 1.8 mm separation in elevation. However, significant artifacts in the temperature maps were observed above a separation of 0.7 mm due to localized decorrelation.

As the speed of sound in the heated region is changed, the heated tissue can act as an aberrator for the imaging system. The decreased correlation between the reference frame and later frames, observed while heat was generated (*t* = 10 s to *t* = 40 s) is related to this thermo-acoustic lens effect [31, 43].

### Application: mild hyperthermia using thermal strain

**Fig 9:**
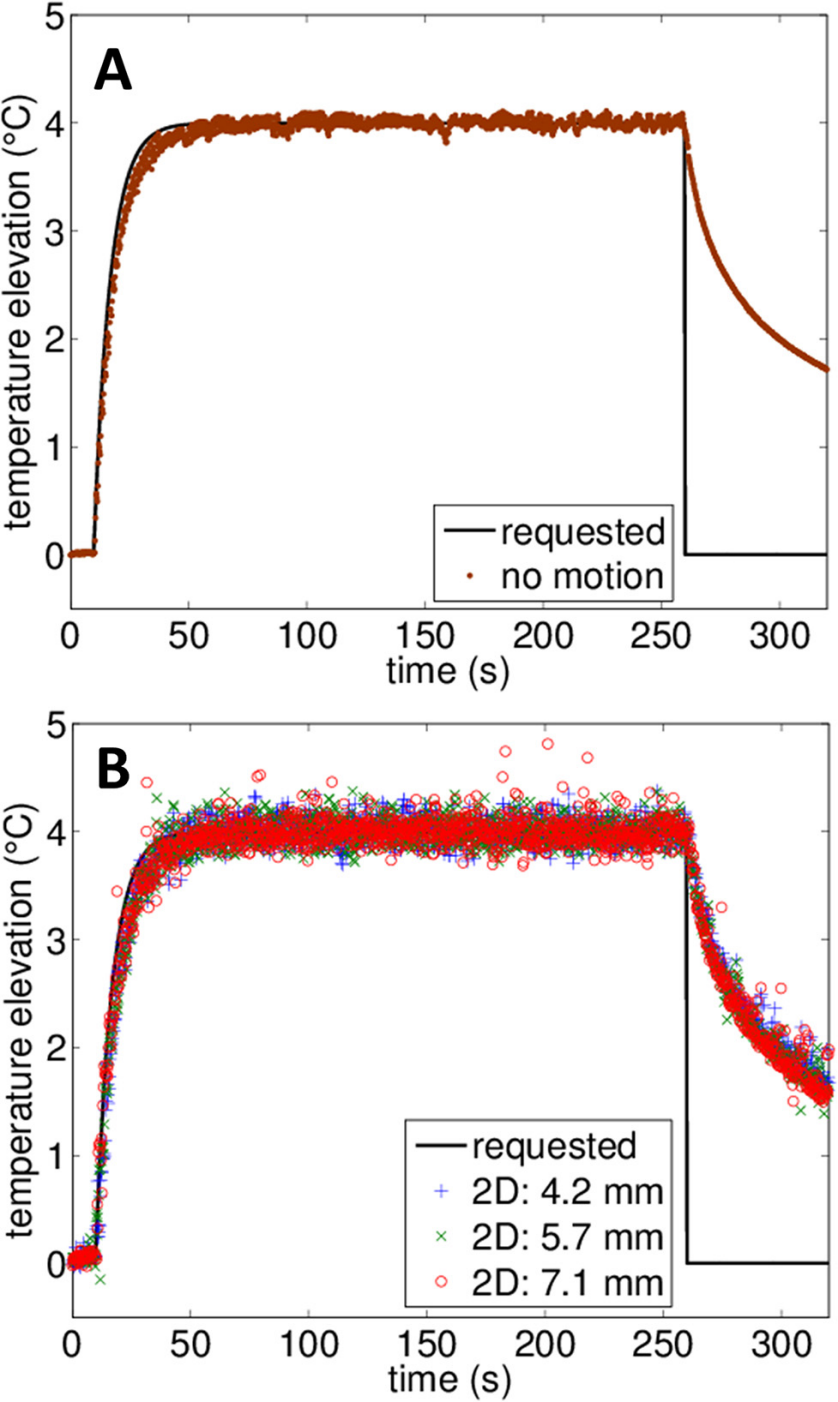
PID control of temperature elevation using strain-based temperature measurement. Comparison between requested temperature and strain-based temperature during PID control of hyperthermia (A) without motion and (B) in the presence of 2-D periodic motion with different displacements.

The 2-D motion pattern tested in the previous section was used to evaluate thermal strain as the feedback for a PID controller and was first tested without probe motion. As displayed in Fig 9A, tracking of the temperature was successfully achieved and temperature was maintained within 0.1°C of the requested +4°C increase in the steady-state. In this case, the standard deviation of the temperature was smaller than 0.1°C which demonstrates the high accuracy of the thermal strain estimation. The slow temperature rise was sufficient to smooth the system response and no oscillations were observed during the experiments. The 4 Hz framerate allowed a sufficient flow of feedback information for an accurate control of temperature. At the end the heating (t = 260 s), the magnitude of the correlation over the ROI reached a minimum value of 0.87 in relation with the thermal lens effect.

With 2-D motion and 4.2 mm displacement of the imaging probe (**Fig 9B**), control of hyperthermia was similarly achieved and the error was less than 0.3°C. Similar results were found with larger displacements, although a few isolated time points showed an error greater than 0.5°C for the 7.1 mm displacement experiment but had no effect on the temperature control. These results validate the robustness of the motion compensation method and demonstrate the possibility of non-invasively controlling the temperature using ultrasound imaging.

## Discussion

The immediate significance of creating a system for ultrasound-generated hyperthermia is driven in part by cancer treatment therapies involving temperature-sensitive drug carrying particles [2, 3]. Despite being currently under pre-clinical and clinical testing, their translation is limited by the availability of systems for controlled hyperthermia. By combining temperature-sensitive particles with hyperthermia, a large fraction of drug can be delivered into tumors without systemic toxicity [3]. Mice with aggressive local syngeneic tumors have been cured: every tumor cell was eliminated and 100% of mice survived more than 1 year after treatment ended without evidence of cancer in a protocol with minimal toxicity that would not be possible with free drug or available commercial therapeutics [3]. Several expected advantages of this approach were reported [3, 44–46]: 1) the drug does not need to accumulate via the enhanced permeability and retention effect, instead it was released in the tumor vasculature and accumulates in part in endothelial cells; 2) depending on the size of the region of release and the time of activation, accumulation can be far greater than typical cancer nanotherapeutics and 3) the released drug can be rapidly internalized.

MRI-guided focused ultrasound (MRgFUS) is currently undergoing clinical trials for a broad range of ablative applications (e.g. uterine fibroid ablation [47] and palliative treatment of bone metastasis [48]) and mild hyperthermia was also investigated under MR guidance [15, 16]. Due to cost and accessibility, MR-guidance may limit adoption of this potentially curative technology. The use of MRgFUS for guiding drug delivery is impractical as such devices are not portable and are not available in the infusion clinics used to administer chemotherapy. Therefore, the creation of an ultrasound-guided system is particularly timely. An integrated ultrasound imaging and therapy system is required for hyperthermia because temperature must be estimated and controlled with an integrated control loop (as is done in MRgFUS). An integrated imaging and therapy system will also maximize the frame rate since the therapeutic and imaging beams must be efficiently interleaved.

Similar strain-based temperature control has been previously reported for samples in the absence of motion [18, 20]. To the best of our knowledge, this represents the first paper to explore the limits on non-invasive temperature control of mild hyperthermia in presence of motion. The ability to spatially and temporally monitor temperature is of great interest to keep track of the thermal dose and thus have an accurate estimation of the CEM43 metric [9].

In this work, real-time ultrasound thermometry with motion compensation was successfully tested on a tissue mimicking phantom. Temperature estimation was shown to be robust to tissue motion even in the presence of out-of-plane or aperiodic motion. The system’s sensitivity to changes in temperature was similar with and without motion and the RMSE was smaller than 0.3°C. The method applied here can provide accurate spatial and temporal resolution of the temperature elevation and may be of great interest for localized mild hyperthermia application such as delivery of temperature sensitive drug carriers to tumor site [3].

Recent studies employing ultrasound-based temperature estimation with motion compensation have reported *in vivo* results with rodents. In the study from Bayat *et al*. [30], a high frame rate was required to maintain the correlation between adjacent successive frames (91 frames/s). The technique is based on estimating the incremental time-shift between two successive frames. In the particular application of mild hyperthermia, the heating rate is low (approximately 0.1°C/s or lower) to ensure that thermal damage does not occur in the targeted tissue [15, 16]. In this case, a method based on incremental time-shift may not be adequate to monitor the slow temperature rise targeted in mild hyperthermia. The present work estimates the time shift with baseline images acquired before heating and therefore measures the temperature change compared to a reference value.

The study from Seo *et al*. [21] reports temperature estimation using the change in ultrasound backscattered energy on tumor-bearing mice using the same imaging array used in this study (L7-4) and a similar multi-reference frame approach. The decorrelation between frames caused by the mouse motion appears to be relatively small and 150 references frames were recorded. In the present work, complete decorrelation was found between frames during 3-D motion and the algorithm correctly estimated the temperature. Based on that result, the present algorithm is expected to provide robust and accurate temperature feedback when tested on small animals. In addition, we have compared our results to the change in backscattered energy as described in Seo *et al*. study on the sequences described in this work. Under these conditions, meaningful temperature estimates could not be generated in the presence of motion.

Other techniques for temperature estimation can be explored in the future. Using shear modulus changes with temperature, Arnal *et al*. were able to monitor temperature with a low sensitivity to tissue motion with a reported noise of 0.25°C in presence of motion [33]. This value is similar to the RMSE found in this work. For the high resolution *in vivo* applications considered here, shear wave thermometry was not tested due to the spatial resolution required for preclinical studies and for treating small tumors. Spatial compounding was proposed by Pernot *et al*. to improve temperature estimation and reduce the thermal lens artifacts appearing behind the heated region [49]. This method could be implemented to further enhance the temperature estimation method presented in this work.

There were several limitations to the study performed here. Although thermal strain estimation was shown to be accurate in presence of linear translation or axial compression, the effect of more complex motion (such as rotation) or other forms of deformation (lateral compression, warping) was not tested and the present algorithm may not be sufficient to compensate for such motion. Further work will focus on testing the present method on *in vivo* data. The phantom used in this study was calibrated and homogeneous throughout the whole volume. Application to heterogeneous tissues or tumors may be challenging but may also provide the opportunity to track distinct structures.

The motion compensation algorithm presented in this work is based on the hypothesis of physiological motion that can be spatially sampled by multiple reference frames. In this case, the temperature was shown to be independent of motion periodicity but dependent on the correlation between frames. If the tissue displacement is outside of the boundaries represented by the reference frames during treatment, correlation between frames may be lost and thermal strain estimation will thus perform poorly. In such a case, a threshold on the correlation value between frames may be used as a metric to discard uncorrelated data.

The non-co-linear configuration of the therapeutic and imaging array tested in this work is not a practical configuration for *in vivo* work. A co-linear system is currently in construction. It was reported that this configuration was noisier in the presence of a sharp lateral temperature gradient [50]; however, the 2-D tracking method which is used here reduces such noise. Further, in mild hyperthermia the temperature gradients are reduced. Thus, the co-linear configuration is not expected to significantly impact the performance of the method.

## Conclusion

In this work, accurate estimation of temperature elevation was achieved in a tissue-mimicking phantom in the presence of motion. Under the assumption of periodic motion, a speckle tracking algorithm based on 2-D normalized cross-correlation was used to estimate thermal strain using a buffer of reference frames which sample the motion in space. Motion compensation was successfully tested for motion patterns (diagonal, axial compression) that were designed to be similar to physiological motion and with both periodic and aperiodic motion. After optimization of the algorithm, real-time display of the temperature map was achieved at 4 fps. The results indicate that the accuracy of the temperature estimates and the frame rate were sufficient to accurately monitor and control hyperthermia using a PID controller. Thus, this method seems promising to control hyperthermia with accurate spatial and temporal feedback while being completely non-invasive. Studies of performance in the presence of more complex motion and extensions to a multi-frequency array are underway.

## Supporting Information

S1 Fig Periodic and aperiodic motion tested for the 2-D diagonal motion. The position indicates the position of the imaging array along the diagonal trajectory. For the periodic motion, the total displacement is 7.1 mm and reaches a maximum speed of 7.1 mm/s. The aperiodic motion has random positions along the diagonal and is limited to a 7.1 mm displacement range.
(TIF)

S2 Fig Decorrelation of ultrasound data as a function of elevation (out-of-plane) distance measured for the L7-4 imaging array. Each point of the curve indicates the magnitude of correlation with a reference frame (elevation = 0 mm).
(TIF)

S1 Movie Temperature map and ultrasound imaging with axial compression. The imaging probe applies a periodic axial compression on the phantom (2 s periodicity) reaching a maximum strain of 5% (2.5 mm displacement).
(AVI)

S2 Movie Temperature map and ultrasound imaging with 2-D motion. The imaging probe is cyclically translated in the imaging plane (2 s periodicity) for a total displacement of 4.2 mm.
(AVI)

S3 Movie Temperature map and ultrasound imaging with 3-D motion. The imaging probe is cyclically translated with combined in plane and out-of-plane motion (4 s period) for a total displacement of 5.2 mm.
(AVI)
