# Supplementary figures and images for: Spatial and Temporal Control of Hyperthermia Using Real Time Ultrasonic Thermal Strain Imaging with Motion Compensation, Phantom Study
Source: PLoS One. 2015 Aug 5;10(8):e0134938. doi: 10.1371/journal.pone.0134938 (PMC4526517; doi:10.1371/journal.pone.0134938)

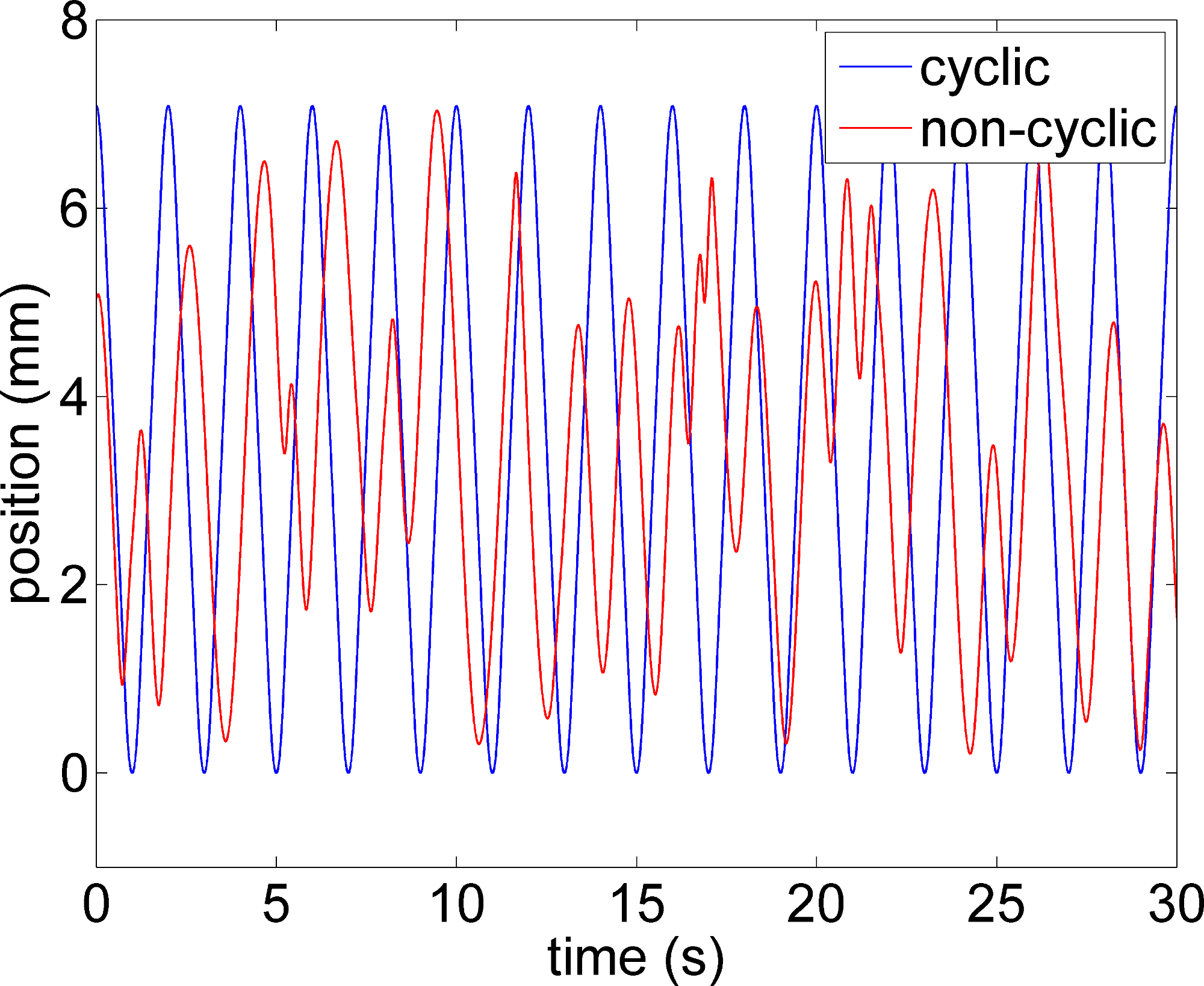

Supplement: S1 Fig — The position indicates the position of the imaging array along the diagonal trajectory. For the periodic motion, the total displacement is 7.1 mm and reaches a maximum speed of 7.1 mm/s. The aperiodic motion has random positions along the diagonal and is limited to a 7.1 mm displacement range. (TIF) [file pone.0134938.s001.tif]

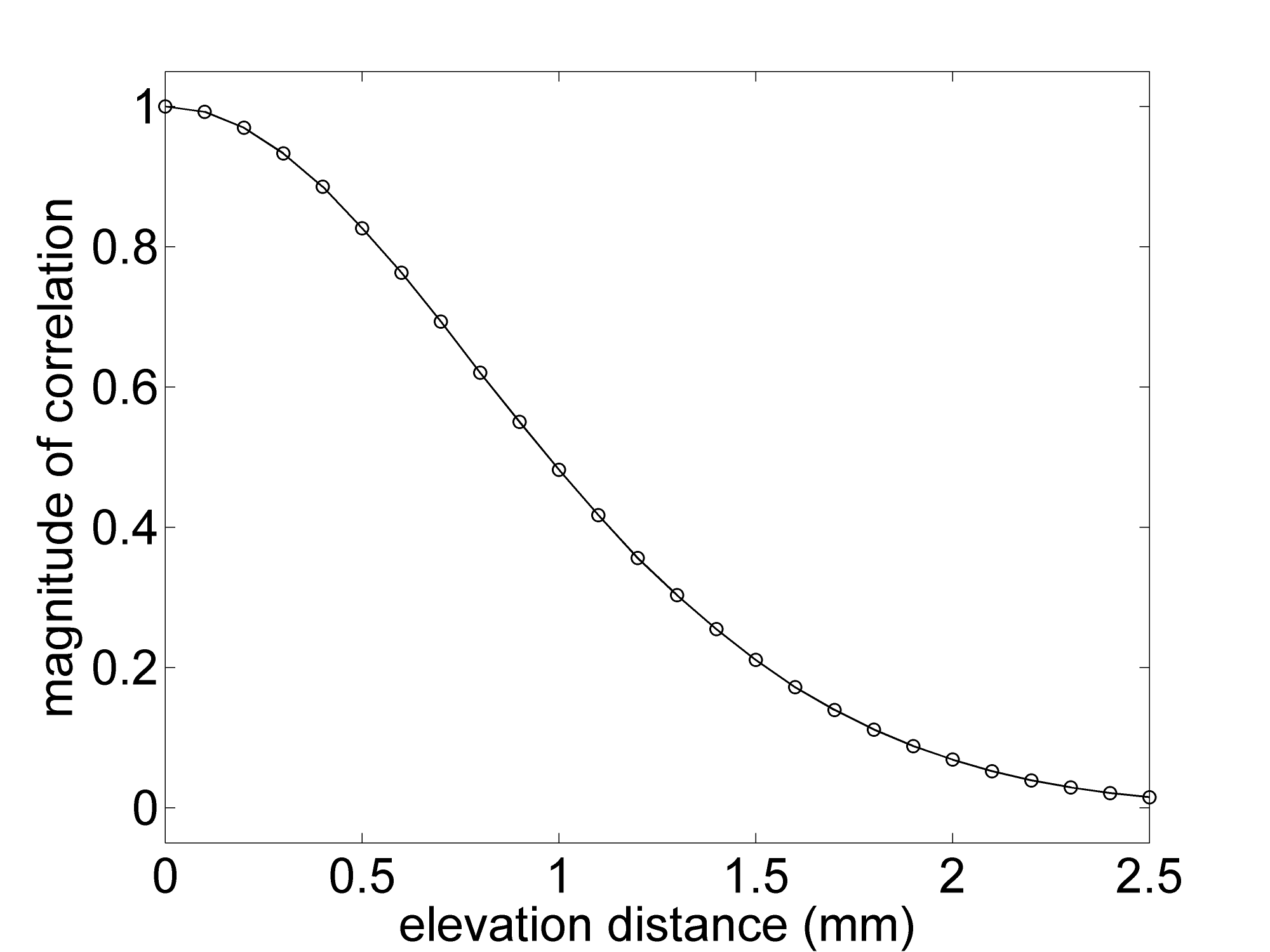

Supplement: S2 Fig — Each point of the curve indicates the magnitude of correlation with a reference frame (elevation = 0 mm). (TIF) [file pone.0134938.s002.tif]
